# Healthcare professionals’ knowledge and views on unmet needs in prevention of infectious comorbidities in pregnant women with inflammatory rheumatic diseases

**DOI:** 10.1007/s00296-026-06174-5

**Published:** 2026-06-15

**Authors:** Dinara Yerlanova, Olena Zimba, Dinara Makhanbetkulova, Bekaidar Nurmashev, Mariusz Korkosz, Burhan Fatih Kocyigit

**Affiliations:** 1 Department of Chemical Disciplines, Biology and Biochemistry, South Kazakhstan Medical Academy, Shymkent, Kazakhstan; 2 Department of Rheumatology, Immunology and Internal Medicine, University Hospital in Kraków, Kraków, Poland; 3 National Institute of Geriatrics, Rheumatology and Rehabilitation, Warsaw, Poland; 4 Department of Internal Medicine N2, Danylo Halytsky Lviv National Medical University, Lviv, Ukraine; 5 Department of Nursing, Asfendiyarov Kazakh National Medical University, Almaty, Kazakhstan; 6 Department of Rheumatology and Immunology, Jagiellonian University Medical College, Kraków, Poland; 7 Department of Physical Medicine and Rehabilitation, University of Health Sciences, Adana City Research and Training Hospital, Adana, Türkiye

**Keywords:** Rheumatic diseases, Pregnancy, Prenatal care, Infections, Surveys and questionnaires, Cross-sectional studies, Health knowledge, attitudes, practice

## Abstract

Inflammatory rheumatic diseases (IRDs) present substantial risks of infection-related comorbidities during pregnancy. This study evaluates the knowledge, experience, and perceptions of healthcare professionals concerning the prevention of these risks. An international, cross-sectional survey was administered using the SurveyMonkey platform. The survey was disseminated to healthcare professionals specializing in rheumatology, obstetrics, infectious diseases, internal medicine, general practice, and related disciplines through social media channels. Developed in accordance with European Alliance of Associations for Rheumatology (EULAR) recommendations, this survey comprised 30 questions, including multiple-choice, Likert-type, and open-ended formats. A total of 201 healthcare professionals from thirty-six countries participated in the study, with rheumatologists comprising the majority (*n* = 145, 72.1%). Systemic lupus erythematosus (*n* = 183), systemic vasculitis (*n* = 141), and rheumatoid arthritis (*n* = 86) were identified as the diseases associated with the highest risk of infection-related comorbidities. The most frequently recommended infectious conditions for screening included Hepatitis B (*n* = 142), urinary tract infections (*n* = 141), and Hepatitis C (*n* = 129). The primary risk factors were uncontrolled disease activity (*n* = 176), high-dose corticosteroid use (*n* = 164), and high disease severity (*n* = 162). The most significant systemic barriers were insufficient number of specialists (*n* = 156), and absence of multidisciplinary teams (*n* = 155). This study identifies structural and educational deficiencies in the management of infection-related comorbidities among pregnant patients with IRD. The results underscore the need for targeted clinical guidelines, enhanced multidisciplinary care models, and expanded pre-pregnancy counseling.

**Supplementary Information:**

The online version contains supplementary material available at 10.1007/s00296-026-06174-5.

## Introduction

Inflammatory rheumatic diseases (IRDs) are chronic and systemic conditions that commonly affect women of reproductive age [1]. Comprehensive pregnancy planning, monitoring, and management are crucial to optimize maternal and fetal outcomes [2]. Inadequate control of disease activity before and during pregnancy increases the risk of preeclampsia, preterm birth, low birth weight, and other obstetric complications [3, 4]. The development of appropriate treatment strategies, effective management of disease activity, and prevention of complications are essential clinical priorities. Additionally, drug selection and immunomodulatory therapy require a multidisciplinary approach to safeguard maternal and fetal health [5, 6]. Consequently, the management of pregnancy in women with IRDs demands close collaboration between rheumatology and obstetrics specialists.

Pregnant individuals with IRDs are at increased risk of infections due to the underlying disease, immunosuppressive therapies, and immunological changes associated with pregnancy [7, 8]. Early diagnosis, prevention, and management of infection-related comorbidities are essential to reduce complications for both mother and fetus [9]. Medications commonly used in the management of IRDs may further elevate infection risk.

Survey-based studies provide a systematic approach to assessing participants’ knowledge, experiences, and perceptions. This methodology enables the collection of data that is more generalizable than that obtained through individual observations, particularly when engaging large participant pools at national or international scales. Additionally, the reliability and comparability of the data are enhanced by collecting responses anonymously and in a standardized manner [10, 11].

This study aims to analyze the perspectives of rheumatologists, obstetricians, infectious disease specialists, and other relevant stakeholders through an international survey. The survey gathers data on current practices, experiences, and perceptions related to the prevention of infection-related comorbidities in individuals with IRDs during pregnancy. Additionally, it seeks to identify unmet needs, risk-assessment strategies, multidisciplinary approaches, and educational requirements in this context. The results are intended to inform the development of practical recommendations for the prevention and management of infection-related comorbidities and may contribute to international guidance on managing IRDs during pregnancy.

## Methods

This international cross-sectional survey aimed to assess the knowledge and views of rheumatologists, obstetricians, infectious disease specialists, general practitioners, internal medicine physicians, and other healthcare professionals about unmet needs in preventing infection-related comorbidities in pregnant patients with IRDs. The survey was arranged via the SurveyMonkey platform, with the link electronically distributed to relevant healthcare professionals and data collected online.

### Survey design

The questionnaire was developed in accordance with European Alliance of Associations for Rheumatology (EULAR) recommendations for managing rheumatic diseases during pregnancy and with EULAR guidelines on screening and preventing infections in adults with these diseases [12, 13]. The following reports were also consulted in designing the questionnaire [14, 15, 16]. This framework ensures the survey’s international relevance, addressing both pregnancy and infection risks. To ensure the accuracy and validity of the survey content, two rounds of review were conducted by five specialists. The survey questions were evaluated for grammatical accuracy, style, and topic coherence. A simulated online form-filling session was subsequently undertaken to evaluate the survey’s practical usefulness. Before the data-gathering procedure, 10 independent healthcare experts from diverse disciplines were invited to complete the survey, and feedback was obtained after evaluating their responses. The survey was amended and completed based on the recommendations received. The questionnaire consists of 30 questions, 13 of which are in a “select all that apply” format, allowing multiple answer options. Three of the questions were answered using a Likert scale, in which participants rated importance with stars from 1 (“not important”) to 5 (“extremely important”). One question, “What would you recommend to improve health services to pregnant patients with IRDs and infectious comorbidities?”, was open-ended. The remaining questions were single-choice, and participants were asked to share socio-demographic information, professional experience, and opinions on relevant clinical practices. Participants could modify their responses at any point before submission; however, alterations were prohibited after submission. All questions were mandatory.

### Sampling strategy

The survey was carried out using a convenience sampling approach. The survey link was distributed between January 27, 2026 and March 16, 2026 via X, Facebook, and other social media channels. The preambule of the survey included an informed consent detailing the study’s purpose, data use policies, and participants’ rights. No financial or other incentives were provided to participants, which helped maintain the objectivity of the responses.

### Confidentiality, integrity, and availability

Anonymization measures were implemented in the study to protect participants' privacy. Internet Protocol (IP) addresses and participants' email addresses were used as unique identifiers to ensure each response was unique. The study moderator assured the highest level of data privacy by protecting IP addresses and email information. The collected data were then stored in anonymized spreadsheet. This report followed the design recommendations proposed by Zimba O and Gasparyan AY [17].

### Ethics approval

The survey study protocol was reviewed and approved by the Local Bioethics Committee of South Kazakhstan Medical Academy, Shymkent, Kazakhstan (Protocol N3, February 21, 2024).

### Analysis and data presentation

The results section primarily uses descriptive statistics. Data are presented as numbers (n), percentages (%), and medians (minimum–maximum). Data entry and analysis were performed in Microsoft Excel, and the accuracy and integrity of the data were maintained throughout.

## Results

### Participant characteristics and experience

**Fig. 1:**
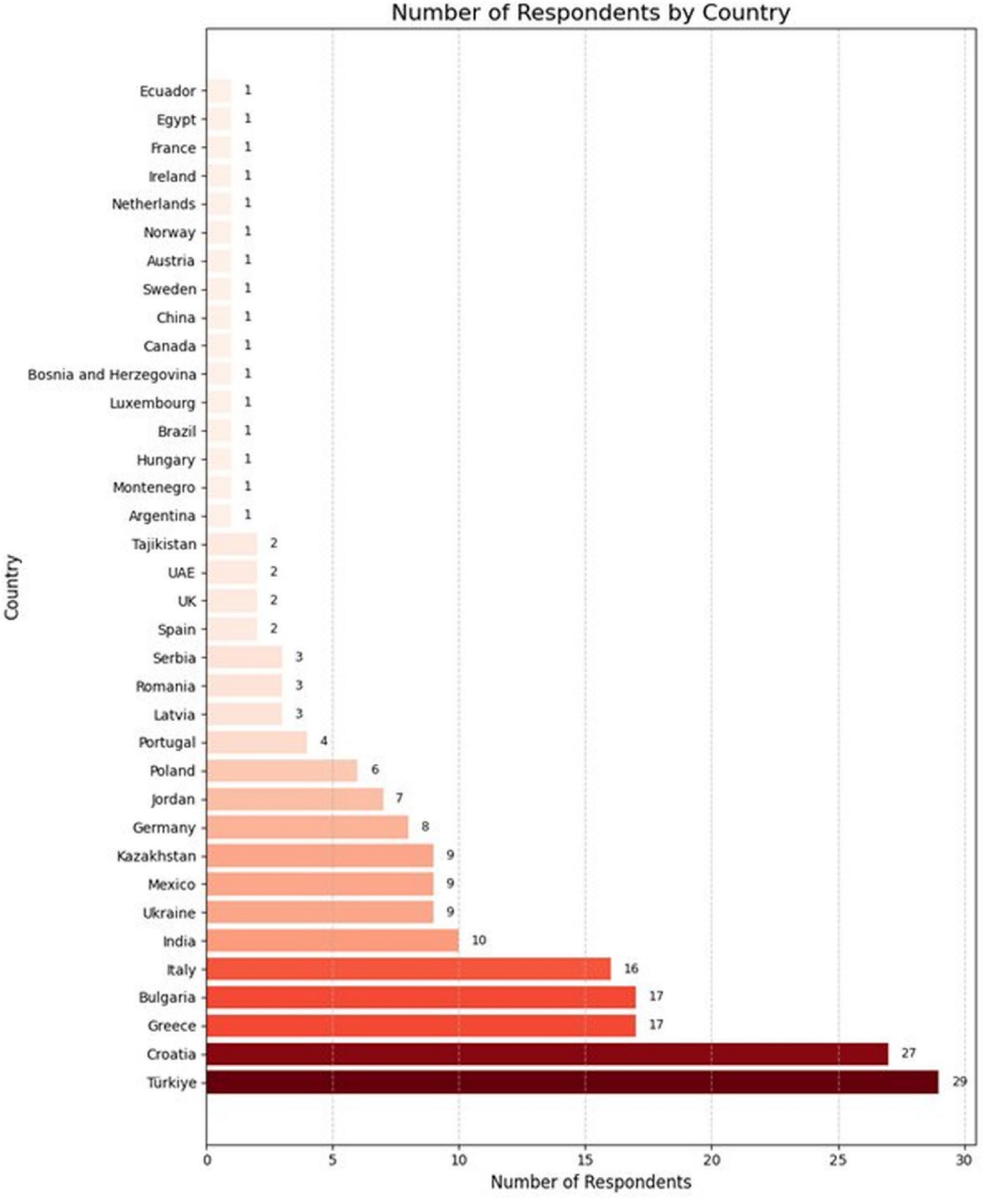
Geographic distribution of survey respondents across 36 countries, indicating the number of responses from each country

**Fig. 2:**
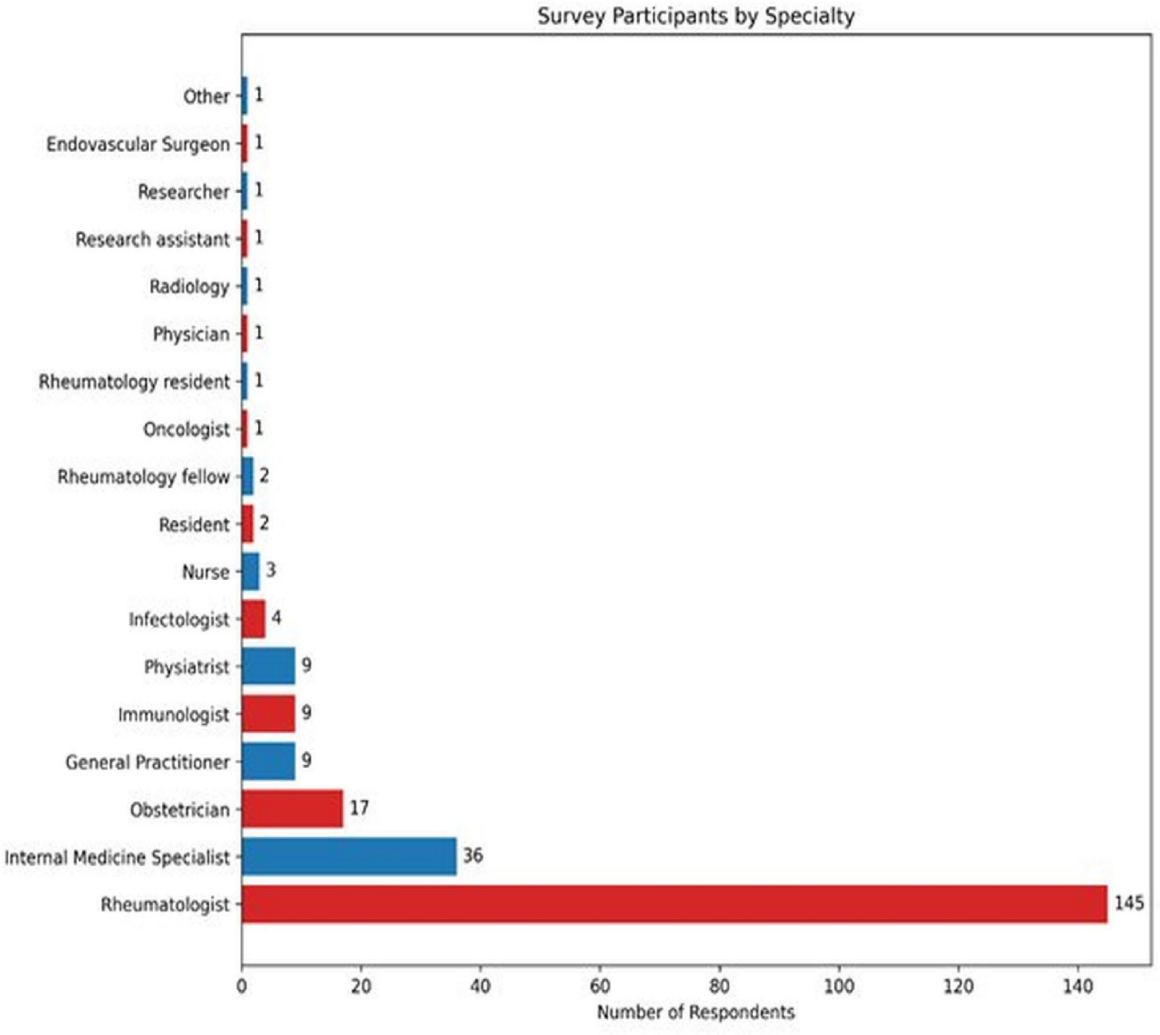
Distribution of survey respondents by specialty

Data were provided by 201 respondents. The median age of respondents was 41 (20–73). Of the participants, 123 (61.2%) were females, 75 (37.3%) were males. Responses were received from 36 different countries. The top five countries by response count were Türkiye (*n* = 29), Croatia (*n* = 27), Greece (*n* = 17), Bulgaria (*n* = 17), and Italy (*n* = 16). The distribution of responses by country is shown in Fig. 1. The three most represented specialties among the participants were rheumatologists (*n* = 145), internal medicine specialists (*n* = 36), and obstetricians (*n* = 17). The distribution of specialties is shown in Fig. 2.

Most participants worked at university-affiliated hospitals (*n* = 121), followed by public hospitals (*n* = 51) and private hospitals (*n* = 21). Other centers included a primary health care centre (*n* = 3), private practices (*n* = 4), and the national research oncology center (*n* = 1). 93.1% of participants (*n* = 187) worked in urban settings, while 6.9% (*n* = 14) worked in rural settings. The participants’ professional experience was categorized as follows: more than 10 years (*n* = 116), 5 to 10 years (*n* = 43), 1 to 5 years (*n* = 38), and less than 1 year (*n* = 4). The median number of pregnant patients with rheumatic diseases that participants examined annually was 10. The median number of pregnant patients with rheumatic diseases and infectious comorbidities that participants examined annually was 3.

### Knowledge and awareness of infectious comorbidities

Upon inquiry regarding resources for further education on infectious comorbidities in pregnant patients with rheumatic diseases, respondents most frequently recommended practice guidelines of global professional societies (EULAR/ACR) (*n* = 181), followed by ClinicalKey and/or UpToDate (*n* = 144), and bibliographic databases such as PubMed, Scopus, Web of Science, or DOAJ (*n* = 112). Other resources included handouts from pharmaceutical companies (*n* = 110), textbooks (*n* = 76), professional congresses, conferences, seminars, and meetings (*n* = 71), artificial intelligence tools (*n* = 43), search engines such as Google Scholar (*n* = 32), social media platforms (*n* = 10), and other sources (*n* = 6).

Most respondents (*n* = 170, 84.6%) were familiar with the updated 2024 EULAR guidelines for using antirheumatic drugs during pregnancy. A smaller group expressed uncertainty (*n* = 15, 7.5%) or reported being unfamiliar with these recommendations (*n* = 16, 7.9%). The frequency of responses regarding discussing the prevention of infectious comorbidities in pregnant patients with rheumatic diseases was as follows: “always” by 54, “often” by 64, “sometimes” by 56, “rarely” by 24, and “never” by 3.

### Risk assessment and disease factors

**Fig. 3:**
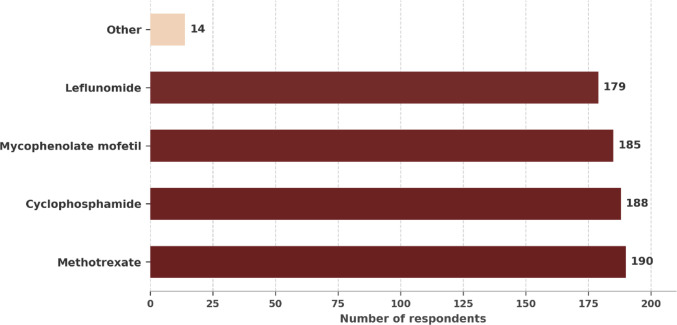
Drugs contraindicated in pregnancy among patients with inflammatory rheumatic diseases

**Fig. 4:**
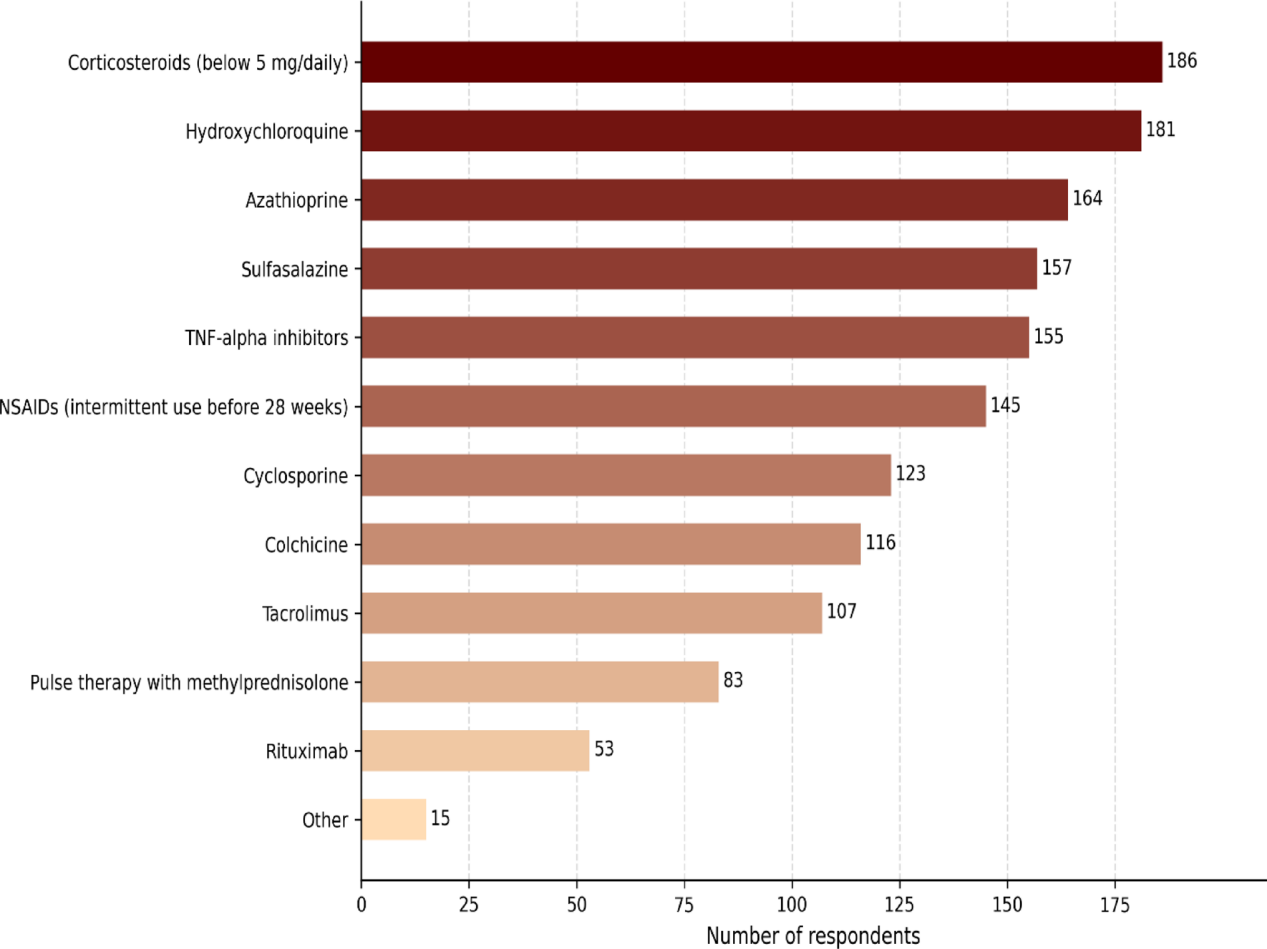
Drugs identified as safe during pregnancy in patients with inflammatory rheumatic diseases

**Fig. 5:**
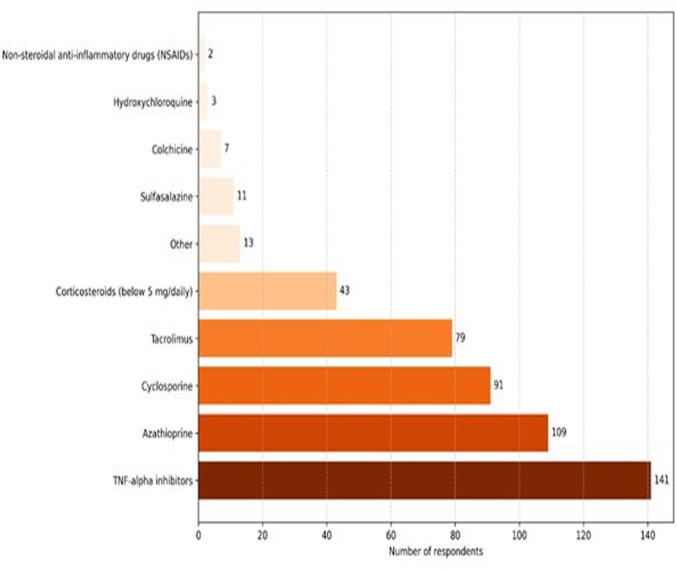
Drugs increasing the risk of infectious complications during pregnancy in patients with inflammatory rheumatic diseases

Participants highlighted methotrexate (*n* = 190), cyclophosphamide (*n* = 188), mycophenolate mofetil (*n* = 185), and leflunomide (*n* = 179) as drugs which should be avoided in pregnant women owing to concerns of maternal and fetal risks (Fig. 3). Participants identified the main drugs deemed safe for pregnant patients with IRDs. The most common of these drugs were corticosteroids (less than 5 mg/day) (*n* = 186), hydroxychloroquine (*n* = 181), and azathioprine (*n* = 164). Additionally, sulfasalazine (*n* = 157), TNF-alpha inhibitors (*n* = 155), and the intermittent use of non-steroidal anti-inflammatory drugs (NSAIDs) until week 28 of pregnancy (*n* = 145) were noted (Fig. 4). According to participants’ responses, the drugs that most increased the risk of infectious complications in pregnant patients with IRDs were TNF-alpha inhibitors (*n* = 141), azathioprine (*n* = 109), and cyclosporine (*n* = 91) (Fig. 5).

**Fig. 6:**
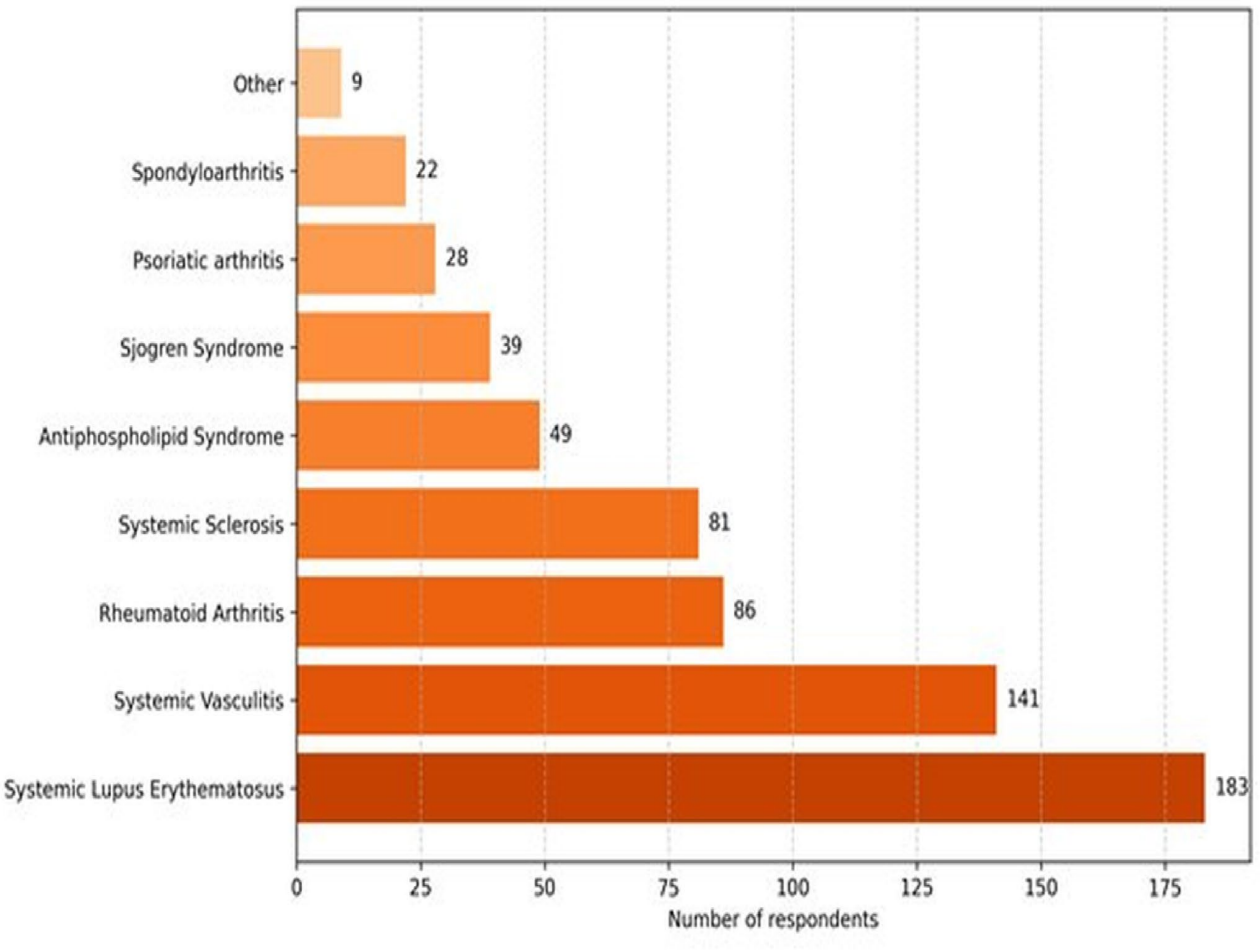
Inflammatory rheumatic diseases with the highest risk of infection-related complications during pregnancy

As shown in Fig. 6, the top three rheumatic diseases reported to have the highest risk of infection-related comorbidities during pregnancy were Systemic Lupus Erythematosus (*n* = 183), Systemic Vasculitis (*n* = 141), and Rheumatoid Arthritis (*n* = 86).

**Fig. 7:**
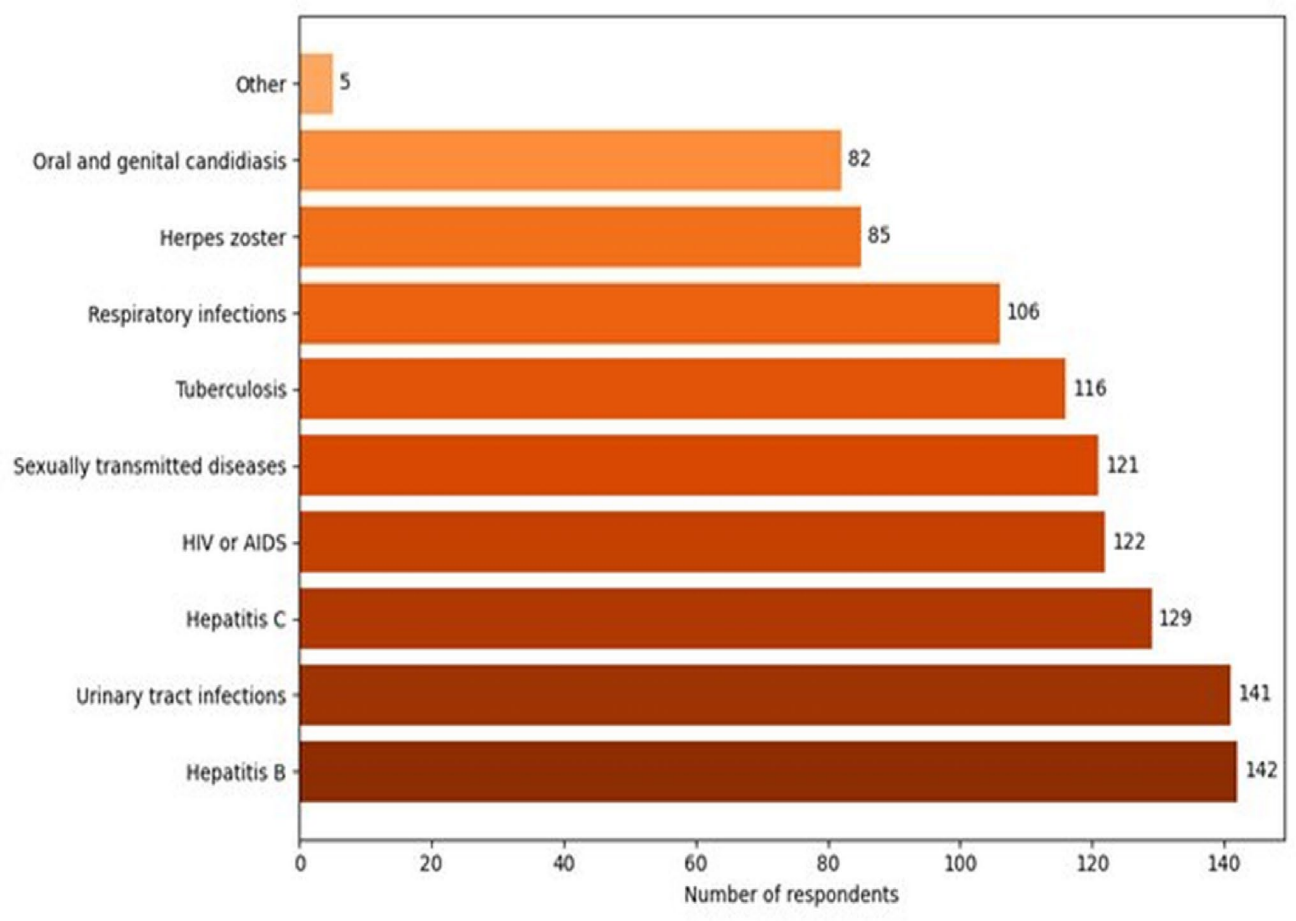
Infectious diseases for proactive screening and prevention in pregnant patients with inflammatory rheumatic diseases

The top three infectious diseases reported by respondents to the survey question, “Which of the following infectious diseases should be proactively screened and prevented in pregnant patients with IRDs ?” were Hepatitis B (*n* = 142), Urinary tract infections (*n* = 141), and Hepatitis C (*n* = 129). The distribution is shown in Fig. 7.

Participants identified multiple factors that increase the risk of infectious comorbidities in pregnant patients with rheumatic diseases. The main risk factors included uncontrolled rheumatic disease (*n* = 176), glucocorticoid therapies at doses above 5 mg daily (*n* = 164), and high disease severity (*n* = 162). 151 respondents highlighted unsafe sexual activities, while non-infectious comorbidities such as arterial hypertension, obesity, and diabetes mellitus were noted by 127 participants. 118 respondents reported smoking, and therapies with disease-modifying antirheumatic drugs were mentioned by 102 participants. 77 respondents identified age above 35 years; and the use of NSAIDs was the least cited risk factor, with 6 participants indicating it.

### Perceived effects of infectious comorbidities on pregnancy outcomes

Respondents evaluated the statement: “Infectious comorbidities increase the risk of adverse pregnancy outcomes in patients with IRDs”. Of them, 83 (41.3%) strongly agreed, 78 (38.8%) agreed, 25 (12.4%) strongly disagreed, 5 (2.4%) disagreed, and 10 (5.1%) remained neutral.

### Multidisciplinary care and professional involvement

Respondents were asked which professionals should be involved in deciding on safe and effective treatment and rehabilitation options for pregnant patients with rheumatic diseases. Most participants highlighted rheumatologists (*n* = 201) and obstetrician-gynecologists (*n* = 194). Other specialists mentioned included infectologists (*n* = 115), immunologists (*n* = 62), nephrologists (*n* = 47), and dermatologists (*n* = 25).

Respondents were asked about the optimal frequency of examinations or pregnancy counselling to diagnose and prevent infectious comorbidities in pregnant patients with rheumatic diseases. The most common choice was regular health check-ups during active or unstable rheumatic disease (*n* = 168). This was followed by check-ups each trimester of pregnancy (*n* = 154) and additional check-ups during corticosteroid and biologic therapies (*n* = 140). Fewer respondents favored check-ups in the first and last trimesters (*n* = 17) and other approaches (*n* = 6).

Participants were queried regarding the rehabilitation strategies for pregnant patients with rheumatic diseases in remission. The most commonly endorsed strategy was physical exercise, encompassing regular aerobic activity (*n* = 152). Other strategies cited included yoga therapy (*n* = 128), Kegel exercises or pelvic floor muscle training (*n* = 119), Pilates (*n* = 99), and physiotherapy (*n* = 92).

The median importance rating for regular pregnancy counselling by multidisciplinary teams in preventing and managing infections was 5 (2–5). The median importance rating for nurses’ and midwives’ involvement in multidisciplinary teams to effectively prevent and manage infections in pregnant patients with rheumatic diseases was 5 (2–5).

### Training and education for healthcare professionals

Respondents were asked if their medical facility or institution offers training in preventing and controlling infectious comorbidities in pregnant patients with IRDs. Of them, 116 declared that no such training is provided, 47 reported that training is available, and 38 were unsure. The survey asked participants to identify activities that could enhance health professionals’ knowledge and skills concerning infectious comorbidities in pregnant patients with IRDs. Continuing medical education (CME) courses on infections in pregnancy were the most frequently selected activity (*n* = 178). Other frequently indicated strategies included interdisciplinary meetings at local medical centres (*n* = 156), regular examinations or consultations for pregnant women with infections (*n* = 133), in-person attendance at international professional congresses (*n* = 114), academic mobility through visits to medical centres specializing in infections during pregnancy (*n* = 107), webinars (*n* = 106), and in-person attendance at local professional congresses (*n* = 102).

Participants rated the significance of enhancing knowledge of infectious comorbidities in pregnant patients with rheumatic disorders for effective infection prevention and management on a scale from 1 to 5, with 1 indicating not important and 5 indicating extremely important. The median rating was 5 (1–5).

### Barriers to effective prevention

Respondents identified healthcare system limitations as key barriers, including the limited number of health professionals with knowledge and skills in infectious comorbidities (*n* = 156), absence of multidisciplinary teams (*n* = 155), and lack of specifically designed practice guidelines for managing infectious comorbidities in pregnant patients with rheumatic diseases (*n* = 146). Additionally, the scarcity of scientific evidence supporting prevention and treatment (*n* = 77) further hampers effective care.

Respondents were asked about how helpful teleconsultations are in preventing and managing infectious comorbidities in pregnant patients with rheumatic diseases. Of the participants, 144 (71.7%) reported they are helpful to some degree, 45 (22.4%) found them helpful, and 12 (5.9%) did not find them helpful at all.

### Open-ended recommendations for service improvement

According to the open-ended suggestions for enhancing health services for pregnant patients with IRDs and infectious comorbidities, the most frequently addressed were strengthening or establishing multidisciplinary teams (*n* = 45), implementing education and awareness initiatives for healthcare professionals and patients (*n* = 38), and improving preventive approaches and counselling, including preconception counselling, vaccination, and risk assessment (*n* = 32).

## Discussion

This international survey identified key findings regarding the management of infection-related comorbidities in pregnant women with IRDs. Respondents reported that insufficient control of disease activity increases the risk of preeclampsia, premature birth, and low birth weight. The study underscored the necessity of multidisciplinary care, particularly collaboration among rheumatologists, obstetricians, and infectious disease specialists. Regular health checkups, trimester-based follow-up, and additional examinations during immunosuppressive treatment were considered essential. Respondents also emphasized the need for education and awareness initiatives, including CME courses, interdisciplinary meetings, and teleconsultation. The absence of guidelines and inadequate multidisciplinary infrastructure were identified as significant barriers.

Responses were collected from 36 countries. The highest participation was from Türkiye (*n* = 29), Croatia (*n* = 27), Greece (*n* = 17), Bulgaria (*n* = 17), and Italy (*n* = 16).The most common specialties among participants were rheumatologists (*n* = 145), internal medicine specialists (*n* = 36), and obstetricians (*n* = 17). The predominant presence of rheumatologists is significant because they serve as the primary clinicians responsible for disease management in this patient population, and their observations carry direct therapeutic relevance [18]. The participant profile enhances the international generalizability of the findings [19].

The educational resource preferences of participants indicate a prioritization of global professional guidelines (EULAR/ACR) regarding infectious comorbidities in individuals with IRDs during pregnancy.This approach aligns with the complexity and high-stakes nature of managing this patient population [12, 20]. Most participants were familiar with current EULAR pregnancy guidelines and routinely addressed infection prevention, indicating strong professional awareness and effective guideline use in multidisciplinary care. However, nearly 15% of respondents reported unfamiliarity with these practice guidelines. This highlights the need for ongoing dissemination and implementation initiatives beyond the initial publication of guidelines to ensure recommendations are adopted and embedded in routine practice across all relevant fields [21].

The results of this study indicate that both drug selection and disease control in patients with IRDs during pregnancy are critical determinants of infection risk. The elevated maternal and fetal risks linked to agents such as methotrexate, cyclophosphamide, mycophenolate mofetil, and leflunomide underscore the necessity for thorough assessment of these medications during pregnancy planning. The strong consensus regarding these drugs, each identified by more than 175 participants, indicates a well-established understanding of teratogenicity and immunosuppressive toxicity that aligns with current EULAR and ACR guidelines [12, 22]. Conversely, maintaining effective disease control with medications considered safe, including low-dose corticosteroids, hydroxychloroquine, and azathioprine, is essential to minimize the risk of infection and promote maternal and fetal safety. These findings indicate that effective drug management and ongoing monitoring of disease activity are essential to minimize the risk of infection and maintain patient safety during pregnancy [23, 24, 25, 26].

The survey results underscore the critical importance of multidisciplinary approaches for managing infection risk in patients with IRDs during pregnancy. Respondents identified the essential involvement of rheumatologists and obstetricians in decision-making and recognized the supportive contributions of infectologists and immunologists. These findings indicate that a multidisciplinary approach is necessary to achieve effective disease control and infection prevention [27, 28].

The observation that most participants did not receive institutional training on infections in pregnancy highlights potential knowledge gaps and variability in clinical practice. Such disparities may impede the standardization of clinical protocols for the early diagnosis and prevention of infection risks. The training strategies proposed by the participants, including CME courses and interdisciplinary meetings, may facilitate knowledge transfer and promote multidisciplinary interaction [29, 30].

The frequency of systemic barriers, such as workforce deficiencies, lack of multidisciplinary frameworks, and absence of disease-specific guidelines, indicates that the primary challenge in this field is not a lack of knowledge but rather the failure to translate existing knowledge into practice. This distinction holds therapeutic significance, as it implies that solutions are more likely to be found in reorganizing care delivery and improving the training and coordination of experts rather than in conducting new research [31].

The respondents' references to preconception counseling and immunization demonstrate a clinician-driven consensus that effective prevention should begin before pregnancy, in accordance with current EULAR guidelines [12, 13].

### Limitations

This study has some limitations. Using a convenience sample and distributing the survey through social media may have introduced self-selection bias, as those already interested in the topic were more likely to participate. Although participation was global, the geographic distribution of responses was uneven, with most respondents from Türkiye, Croatia, and Greece. This imbalance may limit the generalizability of the findings. The predominant representation of rheumatologists among respondents may have influenced the perspectives collected. The cross-sectional design does not permit causal inference regarding the relationship between identified barriers and patient outcomes. Lastly, the sample size is relatively modest (*n* = 201), which should be considered when interpreting subgroup findings across specialties and countries.

## Conclusion

This international survey study identifies significant gaps in the management of infection-related comorbidities among pregnant patients with IRDs. The results demonstrate that inadequate multidisciplinary team structures, the absence of targeted clinical guidelines, and insufficient training for healthcare professionals constitute major systemic barriers. Effective management should commence in the pre-pregnancy period and depend on coordinated collaboration among rheumatology, obstetrics, and infectious disease specialists. The widespread recognition of teleconsultation as beneficial indicates that digital health tools may help address current structural deficiencies. These findings are intended to inform the development of integrated, evidence-based recommendations and to support the establishment of international guidelines for this patient population.

Large-scale, multicenter studies that provide balanced representation across geographic regions and specialties are required. Prospective cohort studies assessing the impact of multidisciplinary care models on clinical outcomes represent a priority for addressing current evidence gaps. Additionally, qualitative research examining patient perspectives and experiences will complement healthcare professionals’ viewpoints.

## Supplementary Information

Below is the link to the electronic supplementary material.

Supplementary Material 1

Supplementary Material 2

## Data Availability

The primary data supporting this survey can be obtained from the corresponding author upon reasonable request. The authors declare that no part of this report has been copied, published, or submitted for publication elsewhere, in whole or in part.
