# Supplementary material for: Healthcare professionals’ knowledge and views on unmet needs in prevention of infectious comorbidities in pregnant women with inflammatory rheumatic diseases
Source: Rheumatol Int. 2026 Jun 15;46(7):154. doi: 10.1007/s00296-026-06174-5 (PMC13269510; doi:10.1007/s00296-026-06174-5)
Supplement: Supplementary file 1 — Supplementary Material 1 [file 296_2026_6174_MOESM1_ESM.pdf]

Healthcare Professionals’ Knowledge and Views on Unmet Needs in Prevention o Infectious Comorbidities in Pregnant Patients with Inflammatory Rheumatic Diseases

The current international survey is aimed to examine rheumatologists’, obstetricians’, general practitioners’, and internal medicine specialists’ knowledge and views on unmet needs in prevention of infectious comorbidities in pregnant patients with inflammatory rheumatic diseases. The questionnaire will take about 10 – 15 minutes to complete. This questionnaire is designed based on related literature reviews, previous surveys, and relevant practice guidelines.

The questionnaire responses are expected to be based on responders’ knowledge and experience of managing pregnancies in patients with rheumatic diseases. By completing this questionnaire, respondents give their agreement to publicize their anonymized answers. The questionnaire is divided into 4 sections: basic knowledge of infectious comorbidities in pregnant patients with inflammatory rheumatic diseases; perception of the main issues and unmet needs in prevention and management of pregnant patients with inflammatory rheumatic diseases; perspectives of managing pregnant patients with inflammatory rheumatic diseases and infectious comorbidities; socio-demographic information). Although there are some personal demographic and clinical experience questions about responders, all answers will be kept confidential and anonymized and will be used only for research purposes. Only questionnaires with complete answers to all points will be processed.

Thank you in advance for taking your time and sharing your professional knowledge to complete this questionnaire. We greatly appreciate the information you provide to advance health services for pregnant patients with rheumatic diseases and infectious comorbidities.

If you have any questions related to this survey, please do not hesitate to contact the moderator of this survey:
Dr Olena Zimba (zimbaolena@gmail.com)

Healthcare Professionals’ Knowledge and Views on Unmet Needs in Prevention o Infectious Comorbidities in Pregnant Patients with Inflammatory Rheumatic Diseases

Basic knowledge

1. Which of the following resources do you advise for learning more about infectious comorbidities in pregnant patients with rheumatic diseases? Select all that apply.

- [ ] Textbooks
- [ ] ClinicalKey and/or UpToDate clinical information resources
- [ ] Practice guidelines of global professional societies (EULAR/ACR)
- [ ] Recommendations of local professional associations
- [ ] Professional congresses, conferences, seminars, meetings
- [ ] Handouts and other resources of pharmaceutical companies
- [ ] Bibliographic databases and platforms such as PubMed, Scopus, Web of Science, Directory of Open Access Journals (DOAJ)
- [ ] Search engines such as Google Scholar
- [ ] Social media such as X (Twitter), Facebook, and YouTube
- [ ] Artificial Intelligence
- [ ] Other (please specify)

2. Are you familiar with updated recommendations of the European Alliance of Associations for Rheumatology (EULAR) for the use of antirheumatic drugs in pregnancy (2024 update)?

- ○ Yes
- ○ No
- ○ Not sure

3. How often do you discuss prevention of infectious comorbidities with your pregnant patients with inflammatory rheumatic diseases?

- ○ Always
- ○ Often
- ○ Sometimes
- ○ Rarely
- ○ Never

4. Which of the following rheumatic diseases present with the greatest risk of infectious comorbidities in pregnancies? Select all that apply.

- [ ] Rheumatoid Arthritis
- [ ] Systemic Lupus Erythematosus
- [ ] Antiphospholipid Syndrome
- [ ] Systemic Sclerosis
- [ ] Sjogren Syndrome
- [ ] Spondyloarthritis
- [ ] Psoriatic arthritis
- [ ] Systemic Vasculitis
- [ ] Other (please specify)

5. Which of the following infectious diseases should be proactively screened and prevented in pregnant patients with inflammatory rheumatic diseases? Select all that apply.

- [ ] Respiratory infections
- [ ] Tuberculosis
- [ ] HIV or AIDS
- [ ] Herpes zoster
- [ ] Oral and genital candidiasis
- [ ] Hepatitis B
- [ ] Hepatitis C
- [ ] Urinary tract infections
- [ ] Sexually transmitted diseases
- [ ] Other (please specify)

Healthcare Professionals’ Knowledge and Views on Unmet Needs in Prevention o
Infectious Comorbidities in Pregnant Patients with Inflammatory Rheumatic Diseases

Main issues and unmet needs

6. Please choose drug therapies that should be avoided in pregnant patients with inflammatory rheumatic diseases due to maternal and fetal risks. Select all that apply.

- [ ] Cyclophosphamide
- [ ] Mycophenolate Mofetil
- [ ] Methotrexate
- [ ] Leflunomide
- [ ] Other (please specify)

7. Please choose drug therapies compatible with pregnancy in patients with rheumatic diseases. Select all that apply.

- [ ] Hydroxychloroquine
- [ ] Sulfasalazine
- [ ] Tacrolimus
- [ ] Azathioprine
- [ ] Cyclosporine
- [ ] Colchicine
- [ ] TNF-alpha inhibitors
- [ ] Non-steroidal anti-inflammatory drugs (NSAIDs such as ibuprofen intermittent use before 28 weeks of gestation)
- [ ] Corticosteroids (below 5 mg/daily)
- [ ] Pulse therapy with methylprednisolone
- [ ] Rituximab
- [ ] Other (please specify)

8. Please choose drug therapies which confer the greatest risk of infectious complications in pregnant patients with inflammatory rheumatic diseases. Select all that apply.

- [ ] Hydroxychloroquine
- [ ] Sulfasalazine
- [ ] Tacrolimus
- [ ] Azathioprine
- [ ] Cyclosporine
- [ ] Colchicine
- [ ] TNF-alpha inhibitors
- [ ] Non-steroidal anti-inflammatory drugs (NSAIDs such as ibuprofen intermittent use before 28 weeks of gestation)
- [ ] Corticosteroids (below 5 mg/daily)
- [ ] Other (please specify)

9. Which of the following factors increase the risk of infectious comorbidities in pregnant patients with rheumatic diseases? Select all that apply.

- [ ] Uncontrolled rheumatic disease
- [ ] High severity of rheumatic disease
- [ ] Therapies with disease-modifying antirheumatic drugs (DMARDs)
- [ ] Glucocorticoid therapies at doses above 5 mg daily
- [ ] Non-steroidal anti-inflammatory drugs (NSAIDs)
- [ ] Non-infectious comorbidities such as arterial hypertension, obesity, diabetes mellitus
- [ ] Smoking
- [ ] Age above 35 years
- [ ] Unsafe sexual activities
- [ ] Other (please specify)

10. Please mark your agreement with the following statement: Infectious comorbidities increase the risk of adverse pregnancy outcomes in patients with inflammatory rheumatic diseases (preeclampsia, gestational diabetes, miscarriage, preterm delivery, low birth weight).

- ○ Strongly disagree
- ○ Disagree
- ○ Neutral
- ○ Agree
- ○ Strongly agree

Healthcare Professionals’ Knowledge and Views on Unmet Needs in Prevention o Infectious Comorbidities in Pregnant Patients with Inflammatory Rheumatic Diseases

Perspectives

11. Who should be involved in the decision-making process to choose safe and efficient treatment and rehabilitation strategies in pregnant patients with rheumatic diseases? Select all that apply.

- ☐ Rheumatologist
- ☐ Obstetrician-Gynecologist
- ☐ Infectologist
- ☐ Dermatologist
- ☐ Nephrologist
- ☐ Immunologist
- ☐ General Practitioner
- ☐ Patient
- ☐ Family Members
- ☐ Other (please specify)

12. Which of the following examination/pregnancy counselling frequencies are optimal for diagnosing and preventing infectious comorbidities in pregnant patients with rheumatic diseases? Select all that apply.

- ☐ Regular health check-ups in active or unstable rheumatic disease
- ☐ Health check-ups each trimester of pregnancy
- ☐ Health check-ups at first and last trimesters of pregnancy
- ☐ Additional health check-ups during corticosteroid and biologic therapies
- ☐ Other (please specify)

13. Which of the following rehabilitation strategies could be recommended to pregnant patients with rheumatic diseases at remission? Select all that apply.

- [ ] Physical exercise (regular aerobic exercise)
- [ ] Yoga therapy
- [ ] Kegel exercises (pelvic floor muscle training)
- [ ] Physiotherapy
- [ ] Pilates
- [ ] Other (please specify)

14. Does your medical centre/university offer training on preventing and managing infectious comorbidities in pregnant patients with inflammatory rheumatic diseases?

- ○ Yes
- ○ No
- ○ Not sure

15. Which of the following activities could be useful for advancing health professionals' knowledge and skills related to infectious comorbidities in pregnant patients with inflammatory rheumatic diseases? Select all that apply.

- [ ] Continuing medical education (CME) courses on infections in pregnancies
- [ ] Academic mobility with visits to medical centres concerned with infections in pregnancies
- [ ] Interdisciplinary meetings at local medical centres
- [ ] Regular examination/consultations of pregnant women with infections
- [ ] In-person attendance of local professional congresses
- [ ] In-person attendance of international professional congresses
- [ ] Webinars

Please rate the following statements (S. No. 16 to 18) on a scale of 1 to 5 stars, progressively increasing in importance.

1 star - not important
5 stars - extremely important

16. Increasing awareness of infectious comorbidities among pregnant patients with rheumatic diseases is important for effective prevention and management of infections.

☆ ☆ ☆ ☆ ☆

17. Regular pregnancy counselling by multidisciplinary teams is important for effective prevention and management of infections.

☆ ☆ ☆ ☆ ☆

18. Involvement of nurses and midwives in multidisciplinary teams is important for effective prevention and management of infections in pregnant patients with rheumatic diseases.

★ ★ ★ ★ ★

19. Which of the following factors are perceived barriers to effective prevention of infectious comorbidities in pregnant patients with rheumatic diseases? Select all that apply.

- ☐ Lack of multidisciplinary teams
- ☐ Limited number of health professionals with knowledge and skills in infectious comorbidities
- ☐ Scarce scientific evidence to support prevention and treatment of infectious comorbidities
- ☐ Lack of specifically designed practice guidelines on management of infectious comorbidities in pregnant patients with rheumatic diseases
- ☐ Other (please specify)

20. Based on your experience, to what extent are teleconsultations helpful for preventing and managing infectious comorbidities in pregnant patients with rheumatic diseases?

- ◯ Not helpful at all
- ◯ Helpful to some extent
- ◯ Helpful

21. What would you recommend to improve health services to pregnant patients with inflammatory rheumatic diseases and infectious comorbidities?

Healthcare Professionals’ Knowledge and Views on Unmet Needs in Prevention o Infectious Comorbidities in Pregnant Patients with Inflammatory Rheumatic Diseases

Socio-demographic information

22. Your gender

- Female
- Male
- Not specified

23. Your age

0 130

24. Which of the following best indicates your specialty background? Select all that apply.

- Rheumatologist
- Obstetrician
- General Practitioner
- Internal Medicine Specialist
- Infectologist
- Nurse
- Other (please specify)

25. Which country do you practise in?

26. Which of the following best describes your medical centre?

- Public hospital
- Private hospital
- University-affiliated hospital
- Primary Health Care Centre
- Other (please specify)

27. How long have you been practising as a health professional?

- Less than 1 year
- 1-5 years
- 5-10 years
- More than 10 years

28. How many pregnant patients with rheumatic diseases do you see/examine annually? (Report an approximate number based on your practice in the last years)

29. How many pregnant patients with rheumatic diseases and infectious comorbidities do you see/examine annually? (Report an approximate number based on your practice in the last years)

30. Location of your healthcare setting

- Urban
- Rural

Thank you for taking the time to share your professional knowledge with us. We greatly appreciate the information you provide to advance
health services for pregnant patients with rheumatic diseases and infectious comorbidities.

If you have any questions related to this survey, please do not hesitate to contact the
moderator of this survey:
Dr Olena Zimba (zimbaolena@gmail.com)
